# Occurrence and Quantitative Microbial Risk Assessment of Methicillin-Resistant *Staphylococcus aureus* (MRSA) in a Sub-Catchment of the Yodo River Basin, Japan

**DOI:** 10.3390/antibiotics11101355

**Published:** 2022-10-05

**Authors:** Takashi Azuma, Michio Murakami, Yuki Sonoda, Akihiko Ozaki, Tetsuya Hayashi

**Affiliations:** 1 Department of Pharmacy, Osaka Medical and Pharmaceutical University, 4-20-1 Nasahara, Takatsuki 569-1094, Japan; 2 Center for Infectious Disease Education and Research (CiDER), Osaka University, Techno Alliance C209, 2-8 Yamadaoka, Suita 565-0871, Japan; 3 Nursing Unit, Jyoban Hospital of Tokiwa Foundation, 57 Kaminodai, Jyoban-Kamiyunaga-Yamachi, Iwaki 972-8322, Japan; 4 Department of Breast and Thyroid Surgery, Jyoban Hospital of Tokiwa Foundation, 57 Kaminodai, Jyoban-Kamiyunaga-Yamachi, Iwaki 972-8322, Japan; 5 Department of Gastrointestinal Tract Surgery, Fukushima Medical University, 1 Hikariga-oka, Fukushima 960-1295, Japan; 6 Department of Food and Nutrition Management Studies, Faculty of Human Development, Soai University, 4-4-1 Nankonaka, Osaka 559-0033, Japan

**Keywords:** antimicrobial-resistant bacteria (AMRB), methicillin-resistant *Staphylococcus aureus* (MRSA), hospital effluent, sewage treatment plant (STP), river environment, quantitative microbial risk assessment (QMRA)

## Abstract

The occurrence of *Staphylococcus aureus* (*S. aureus*) and methicillin-resistant *S. aureus* (MRSA) in a sub-catchment of the Yodo River Basin, a representative water system of a drinking water source in Japan, was investigated. The chromogenic enzyme-substrate medium method was used for the detection of *S. aureus* and MRSA by the presence or absence of antimicrobials in the medium for viable bacteria in a culture-based setting. The contributions of *S. aureus* and MRSA from wastewater to the rivers were estimated based on mass flux-based analysis, and quantitative microbial risk assessment (QMRA) was further conducted for *S. aureus* and MRSA in river environments. The mean abundance of *S. aureus* and MRSA was 31 and 29 CFU/mL in hospital effluent, 124 and 117 CFU/mL in sewage treatment plant (STP) influent, 16 and 13 CFU/mL in STP effluent, and 8 and 9 CFU/mL in river water, respectively. Contribution of the pollution load derived from the target STP effluent to river water ranged from 2% to 25%. The QMRA showed that to achieve the established health benchmarks, the drinking water treatment process would need to yield 1.7 log_10_ and 2.9 log_10_ inactivation in terms of infection risk and disability-adjusted life year (DALY) indexes, respectively. These findings highlight the link between medical environment and the importance of environmental risk management for antimicrobial-resistant bacteria in aquatic environments.

## 1. Introduction

Recently, new environmental water pollution problems caused by antimicrobial-resistant bacteria (AMRB) are increasing globally [1,2,3]. The World Health Organization (WHO) has requested that countries develop a Global Action Plan on Antimicrobial Resistance, a framework for an action plan for AMRB [4], and has advocated that comprehensive measures should be taken to assess and resolve the issues involving AMRB, considering their interactions among humans, animals, and the environment based on the basic concept of One Health [5,6,7]. Among these countermeasures, clarification of the pollution status and an environmental risk assessment of AMRB in the aquatic environments is one of the keys for sustainable human development [8].

The emergence and spread of AMRB has become a serious problem for hospitals and other medical institutions, not only because it makes antimicrobial treatment difficult, but also because it increases the risk of epidemics and severe outbreaks of infectious diseases [9]. WHO has published a list of 12 AMRB that require immediate action, including the development of new drugs. Among these AMRB, methicillin-resistant *Staphylococcus aureus* (MRSA) is classified as a high priority bacterium, after carbapenem-resistant *Acinetobacter baumannii*, carbapenem-resistant *Pseudomonas aeruginosa*, and carbapenem-resistant/third generation cephalosporin-resistant *Enterobacteriaceae*, which are a critical priority [4,10].

Previous reports revealed that hospitals and other medical institutions are not the only contributors to AMRB detected in the environment [11,12]. MRSA, such as extended-spectrum *β*-lactamase (ESBL)-producing *Enterobacteriaceae*, has been a cause of nosocomial infections [13], but it is now becoming prevalent even in cities. In addition, MRSA can be detected in the wastewater of sewage treatment plants (STPs) [14]. Meanwhile, several surveys have been conducted in Europe and the United States on *Staphylococcus aureus* (*S. aureus*), including MRSA, as an indicator microorganism in wastewater and river water [14,15]. In Japan, however, *S. aureus* and MRSA have not legally been used as indicator microorganisms, and there have been almost no reported cases for their occurrence in the water environment. Further, according to a previous global clinical survey, the prevalence of MRSA is 5–25% in Europe and the United States, while in Japan it is at least 50%, which is higher than in the rest of the world [16,17,18]. MRSA itself is considered an opportunistic organism, but is spreading to a wider spectrum of society due to the difficulty in treating it with antimicrobials and disinfectants in medical facilities [19]. According to the previous clinical survey, patients who are opportunistically infected with MRSA have a 17% higher mortality rate than non-infected patients [20].

The environmental risks to human health associated with the persistence of AMRB in aquatic environments are concerning. However, it is not easy to quantitatively assess the risk, even though research on the development and application of risk assessment methods is progressing rapidly [21,22,23]. WHO recommends a preventive water quality management approach using Quantitative Microbial Risk Assessment (QMRA) to control microbiological risk factors for water quality guidelines [24]. This approach has been applied for indicator microorganisms [25] and, more recently, for COVID-19 [26]. Furthermore, QMRA of methicillin-susceptible *S. aureus* and MRSA in reclaimed wastewater is recently being conducted as a case study on risk assessment for AMRB [27,28]. Under these circumstances, it would be possible to quantitatively assess the environmental risk caused by AMRB in aquatic environments and the effectiveness of countermeasures to reduce and/or eliminate the risk of applying this method. However, such studies are limited.

Therefore, the aim of this research was to clarify the status of *S. aureus* and MRSA in actual rivers and wastewaters, and to evaluate human health risks based on QMRA from the results obtained from the survey. In order to achieve this objective, we firstly surveyed the year-round abundance of *S. aureus* and MRSA in wastewater from a hospital, the STP that treats the wastewater, and rivers where the treated water is discharged in the Yodo River Basin, which is one of the representative water systems of a drinking water source in Japan. Next, the environmental dynamics of *S. aureus* and MRSA in the study area were evaluated based on mass flux, and then the contributions of various wastewater discharges into the rivers were estimated. Finally, based on the results obtained from the survey, QMRA was conducted for the following two scenarios to discuss the effects of *S. aureus* and MRSA in aquatic environments on human health: (1) a scenario wherein daily exposure was considered through direct use of river water as domestic water; and (2) a scenario in which measures were taken for river water purification.

## 2. Materials and Methods

### 2.1. Sampling

Hospital effluent, STP influent, STP secondary effluent, STP effluent, and river water were collected from the Yodo River Basin, one of the representative water systems of a drinking water source in Japan; it has a population of 17 million (14% of the Japanese population) [29,30,31,32]. In addition, effluent was also collected from an elderly healthcare facility outside the target basin to determine the situation of AMRB in various medical facilities.

The hospital effluent was directly collected from a hospital with 480 beds that handles an average 1200 patients/d. The hospital effluent flows into the municipal sewage, which processes the waste from a population of 370,000 individuals and is introduced into the STP through an inlet, from which the STP influent sample was collected. The influent is treated using a conventional activated sludge (CAS) process to produce STP secondary effluent, and chlorination disinfection (1.2 mg NaClO/L for 15 min) is executed before it is discharged into the river as STP effluent. The river water was collected at a site 1 km downstream from the effluent discharge point, located in a branch river of the mainstream of the Yodo River. In addition, water from the mainstream of the Yodo River (river water as a drinking water source) was collected, 12 km upstream from the previous sampling site, and the river water at this site was used as a drinking water source, which was supplied to a wide area (1,797,000 m^3^/day), including to the study area and neighboring regions. The effluent from the elderly healthcare facility was directly collected from a facility with 150 beds, with an average of 30 patients/d.

The samples were collected four times in different seasons during 2021 and 2022: May (spring), July, October, and January. To avoid dilution and other external effects, the samples were taken on a rain-free day when no rainfall (<1 mm) had been recorded the preceding 2 d [33]. A 100 mL stainless-steel pail sampler was used to collect wastewater and river water samples. Samples were then placed in sterilized glass bottles, and 0.5 g/L anhydrous sodium thiosulfate (>99%; MilliporeSigma, Burlington, MA, USA) was immediately added to mitigate the effects of any residual chlorine [34,35]. Composite samplers could not be installed to obtain the hospital effluent, the wastewater in STP, or the samples from the elderly healthcare facility, and the placement of sampling equipment along the river is prohibited by law. Therefore, manual sampling was carried out at a fixed sampling frequency [31]. All samples were immediately transported to the laboratory in a cooler box (within 2 h), stored at 4 °C in darkness, and processed within 12 h.

### 2.2. Microbials Analysis

The prevalence of *S. aureus* and MRSA was estimated by screening microbes grown on different chromogenic agar media: CHROMID *S. aureus* Elite for *S. aureus* and CHROMID MRSA for MRSA (bioMérieux S.A., Marcy-l’Étoile, France). Bacterial identification was carried out as per the manufacturer’s instructions and using previously described methods [31,36,37,38,39].

An aliquot (1 mL) of water sample was poured onto each agar plate and spread quickly over the surface. Water samples were applied directly to the culture medium without dilution based on prior investigation and previous reports [32,40]. Each plate was then covered with a cover plate and incubated at 37 ± 1 °C for 24 h in the dark. The bacterial species were differentiated by color and morphology of the colony in accordance with the manufacturer’s specifications and those described previously [41,42,43]. These culture conditions are suitable for the detection accuracy of the target microorganisms, including the results of the genetic information analysis [40,42]. The colonies were counted, and the number of bacteria recovered were expressed as colony-forming units per mL (CFU/mL), which were then converted into mean yearly values. If mean CFU were not whole numbers, the values were expressed as the nearest integer after application of the rounding off rule and counted as N.D. (not detected) if the values were <1. The abundance of MRSA was defined as the proportion of resistant *S. aureus* isolates growing on CHROMID MRSA plates to the total number of *S. aureus* growing on CHROMID *S. aureus* Elite agar plates [32,43]. The relative reproducibility values as repeated measurements on the basis of the repeated measurements (*n* = 3) for MRSA and *S. aureus* were 20% and 18%, respectively.

### 2.3. Calculation of Mass Flux and the Hospital Effluent Contributions to the STP Influent and the River Water Environment in the Target Yodo River Basin

To analyze the environmental dynamics of *S. aureus* and MRSA based on mass flux, the mass flux of *S. aureus* and MRSA in the hospital effluent, wastewater in the STP, and river water in the target Yodo River Basin was calculated, and then their contributions to various wastewater discharges into the rivers based on the obtained values estimated.

The mass flux of both bacteria was calculated by multiplying the numbers of colonies (CFU/mL) by the mean annual flow rates of the hospital, STP, and the river water (m^3^/day). The annual mean flow rates were given as 460 m^3^/day for the hospital effluent; 190,000 m^3^/day for the STP; 2,820,000 m^3^/day for the river water; and 17,800,000 m^3^/day for the river water as drinking water source. These flow values were provided by each facility’s management, local government, and administration [40,44]. The contribution of the mass flux of bacteria to each sampling site in the target survey area was estimated on the basis of previous reports for the calculation of the mass flux of environmental pollutants in water environment [31,45,46].

The contribution of the mass flux for each site was performed assuming that the number of microorganisms do not change in the wastewater or the river water during this period, on the basis that the estimated time from the hospital in the target area to the STP and from the STP to the river is within almost one day, and based on previous reports [32,47,48]. The contribution of the mass flux of bacteria in the hospital effluent to the STP influent was calculated by dividing the individual mass flux of bacteria in the hospital effluent by that of the STP influent, and the contribution of the mass flux of bacteria in the STP effluent to the river water was calculated by dividing the individual mass flux of bacteria in the STP effluent by that of the river water.

### 2.4. Quantitative Microbial Risk Assessment (QMRA) Analysis

The risk of infection and disability-adjusted life years (DALYs) per person per year (ppy) for daily exposure to water for domestic use for one year was calculated according to a previous study [27]. Briefly, the risk assessment of skin or bloodstream infections due to water use was performed separately for MRSA and methicillin-susceptible *S. aureus* (MSSA). The dose–response equations for nasal colonization after immersion are the same for MRSA and MSSA; MRSA and MSSA differ in their concentration in water, the probability of skin and bloodstream infection, and the DALY per case of bloodstream infection.

In this study, QMRA was performed as per a method described in a previous study [27], with some modifications. It should be noted that the methodology used in this paper was based on limited data but is currently available. Horizontal gene transfer was not considered because it has negligible effects on the results as per a previous study [27]. The *S. aureus* concentrations in water were assumed to follow a lognormal distribution based on the measurements in this study. The abundance of MRSA (ratio of MRSA concentration to *S. aureus*) was assumed to follow a normal distribution based on the measurements of this study; a two-tailed truncated normal distribution was used to ensure that no randomly selected value was less than 0 or greater than 1 and that the median of the distribution was the arithmetic mean of the measured values. MSSA concentration was calculated by subtracting the MRSA concentration from the *S. aureus* concentration. We considered a scenario in which river water collected as drinking water was used directly (no inactivation in water treatment processes) and scenarios in which 1, 2, 3, and 4 log_10_ inactivation were achieved by the drinking-water treatment processes.

Monte Carlo simulations were used to calculate the annual risk by running the model for 365 d, with each day’s distribution as independent, for a total of 1000 years. The total risk was calculated by summing the risk values of infections and DALYs for MRSA and MSSA. Crystal Ball software (Oracle Corp, Austin, TX, USA) was used for the analysis.

### 2.5. Statistical Analysis

The tested data were analyzed using Microsoft Excel software, and the data are shown as mean values or mean of logarithmic values with their individual standard deviation (SD) values. A paired *t*-test was performed to evaluate the difference in logarithm of the inactivation rates between *S. aureus* and MRSA at *p* < 0.05 as statistical significance.

## 3. Results

### 3.1. Occurrence of S. aureus and MRSA in the Yodo River Basin

**Figure 1:**
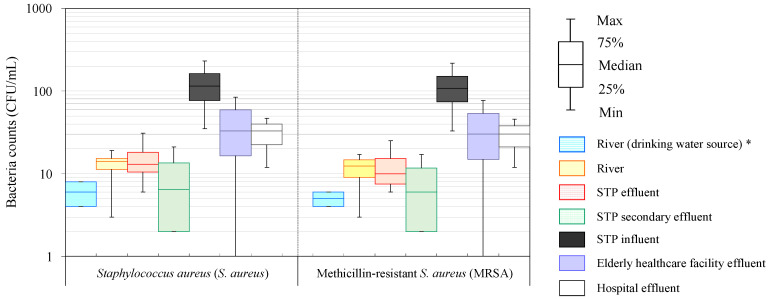
Distribution of *Staphylococcus aureus* (*S. aureus*) and methicillin-resistant *Staphylococcus aureus* (MRSA) in hospital effluent, elderly healthcare facility effluent, sewage treatment plant (STP) influent, STP effluent, and river water (*: The river water as a drinking water source was supplied to the study area and neighboring regions).

*S. aureus* and MRSA were detected during all seasons of the year in the hospital effluent and the STP influent (Figure 1 and Table 1). The mean bacterial counts in the hospital effluent were 31 ± 18 CFU/mL of *S. aureus* and 29 ± 17 CFU/mL of MRSA, while those in the STP influent were 124 ± 83 CFU/mL of *S. aureus* and 117 ± 78 CFU/mL of MRSA. Although there was no statistically significant difference (*p* < 0.05) between the hospital effluent and STP influent for both *S. aureus* and MRSA in this survey, the trends of bacterial counts was lower in the hospital effluent compared to the STP influent, and the mean abundances of MRSA in the hospital effluent and the STP influent were 96% ± 5% and 94% ± 5%, respectively. These bacteria were also detected in the elderly healthcare facility effluent with mean bacterial counts of 39 ± 43 CFU/mL of *S. aureus* and 36 ± 39 CFU/mL of MRSA, and the abundance of MRSA was 94% ± 5%, which was generally consistent with the results in hospital effluent. Similar results in terms of concentration levels and resistance rates in this study were generally reasonable, considering that *S. aureus* and MRSA are microorganisms that tend to be endemic in clinical settings as opportunistic infectious agents [49,50].

*S. aureus* and MRSA detected in wastewater were largely removed in the STP secondary effluent. The mean bacterial counts in the STP secondary effluent were 9 ± 9 and 8 ± 7 CFU/mL for *S. aureus* and MRSA, respectively. The removal rates of *S. aureus* and MRSA due to secondary wastewater treatment, i.e., biological treatment with activated sludge, were 1.03 ± 0.02 log_10_ (93 ± 5%) and 1.03 ± 0.02 log_10_ (93 ± 4%), respectively; however, the difference between the removal rates was not significant (*p* < 0.05). Interestingly, only a small concentration of *S. aureus* and MRSA was removed with chlorine disinfection after biological treatment, and the mean bacterial counts in the STP effluent after chlorination were 16 ± 11 and 13 ± 19 CFU/mL for *S. aureus* and MRSA, respectively, which are similar to the values reported previously (2 × 10^2^ CFU/mL [51,52] and 4 × 10^1^ CFU/mL [31], respectively). The results for the bacterial counts in the STP effluent after chlorination compared to that in STP secondary effluent after biological treatment were slightly higher than those from the manual sampling of wastewater collection, as described in Section 2.1. Chlorine resistance exhibited by *S. aureus* and MRSA is a result of their cell walls, which are stronger than those of other bacteria, rendering them resistant to multiple environmental conditions and even multiple pharmaceuticals [19]. The results were in accordance with those of previous studies considering a previous study, which reported that *S. aureus* and MRSA were gradually inactivated in usual wastewater disinfection process (chlorine injection rate < 15 mg/L) [53].

*S. aureus* and MRSA were also detected in the river water at a level of a few to several CFU/mL. The mean bacterial counts in the river water were 13 ± 7 and 11 ± 6 CFU/mL for *S. aureus* and MRSA, respectively, while those in the river water, which is used as a drinking water source, were 6 ± 2 and 5 ± 1 CFU/mL for *S. aureus* and MRSA, respectively. In addition, the mean abundance of MRSA in the river water (90% ± 12%) was similar to that of wastewater with no significant differences (*p* < 0.05). These results suggest that the *S. aureus* and MRSA would be mainly associated with the wastewater discharged into the river environment around urbanized areas with a high ratio of sewerage, although their origins are diverse [54,55,56].

### 3.2. Mass Flux-Based Analysis of the Contributions of S. aureus and MRSA from the Hospital Effluent to the STP Influent, and from the STP Effluent to the Rivers

The contributions of *S. aureus* and MRSA originating from the hospital effluent to the STP influent in the target basin were analyzed based on mass flux (Table 2). The contributions of *S. aureus* and MRSA from the hospital effluent to the STP influent, and from the STP effluent to the river are shown in Table 3. The contributions of *S. aureus* and MRSA from the hospital effluent to the STP influent based on mass flux ranged from <0.1% to 0.2% and <0.1% to 0.2%, respectively. The contributions of *S. aureus* and MRSA from the STP effluent to the river water and to the river water as a drinking water source ranged from 4.9% to 25% and 3.9% to 25%, and 2.1% to 6.8% and 2.1% to 7.4%, respectively. The contribution of the hospital effluent in polluting the STP obtained in this study was not substantial in the present survey. This could be because the hospitals investigated in this research were one of the medical facilities located within the target STP-covered area, and the volume of the effluent from the target hospital compared to that of the wastewater in the STP was small (<1%).

### 3.3. Quantitative Microbial Risk Assessment of S. aureus and MRSA in the River Environment

For direct use of river water (i.e., no inactivation during water treatment processes), the 95th percentile values were 4.8 × 10^−3^ ppy for risk of infection and 7.8 × 10^−4^ ppy for DALYs (Table 4). These infection risks exceeded the benchmarks, which are often referred to as acceptable risk levels or tolerable burden of diseases for drinking water (10^−4^ ppy [57] for infection and 10^−6^ ppy [58,59] for DALYs). Of these, MRSA accounted for 98.9% of the infection risk and 99.5% of the DALYs, indicating that most of the risk in both indicators was due to resistant bacteria.

**Figure 2:**
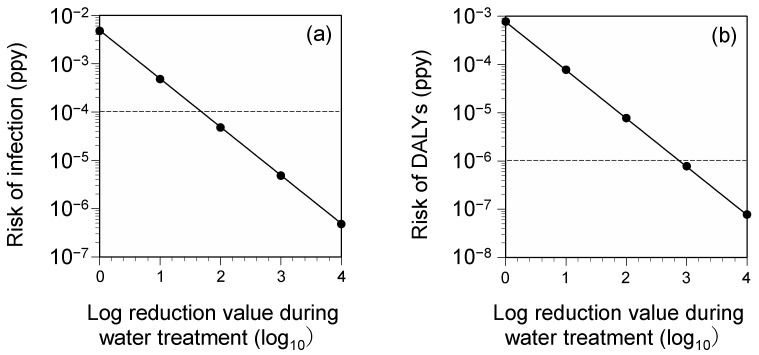
Risk of infection (**a**) and disability-adjusted life years (DALYs) (**b**) 95%tile values are shown. Dotted lines indicate benchmarks.

The risk of infection and DALYs showed a linear reduction with inactivation during the drinking-water treatment process (Figure 2). However, to achieve the established benchmarks, it was calculated that the water treatment process would need to achieve 1.7 log_10_ and 2.9 log_10_ inactivation in terms of infection risk and DALYs, respectively. In the water purification process, the concentration-time (CT) value (mg∙min/L) of free chlorine required for a 3 log_10_ inactivation reduction is approximately 0.5 [60], which is considered to be a sufficiently achievable level for water treatment in Japan, where chlorine-free treatment is generally applied.

## 4. Discussion

The occurrence of *S. aureus* and MRSA in rivers and wastewaters, and the high level of resistance rates, should be considered in light of the prevalence of AMRB in clinical settings as follows. According to Japan Nosocomial Infections Surveillance (JANIS) conducted by the Ministry of Health, Labor and Welfare for medical facilities in Japan, cases of infections with MRSA were reported in 2020 from more than 99.8% of the hospitals and from approximately 2200 hospitals in Japan from 2016 to 2020 [61]. The reported resistance rates of *S. aureus* to the major antimicrobials in inpatients and outpatients as of 2020 are 19% and 35% for clarithromycin, 51% and 80% for erythromycin, 28% and 35% for gentamicin, 52% and 89% for levofloxacin, and 10% and 19% for minocycline. On the other hand, the reported values in that of MRSA were 10% and 26% (MRSA) for clarithromycin, 40% and 77% for erythromycin, 25% and 34% for gentamicin, 36% and 83% for levofloxacin, and 5% and 14% for minocycline, respectively [61]. *S. aureus* and MRSA detected in wastewater and river water in this study could be linked to the recent prevalence of AMRB in clinical settings [62]. Therefore, higher values for the abundance of MRSA are considered to be reasonable, given that MRSA causes nosocomial infections and is thought to be related to the fact that it is becoming widespread not only in hospitals but also in cities [14,62,63].

Ascertaining the current status of *S. aureus* and MRSA in the water environment is essential not only for assessing the problem of water contamination by AMRB, but also for assessing the human health risk from the QMRA. On the other hand, the biochemical and molecular analysis of colonies expressed on culture media for a highly accurate quantitative evaluation of detailed pathogenicity and the health risk assessment of AMRB are considered the important issues [64,65]. In addition, according to the latest reports, the number of cases reported in clinical settings in Japan is still low, but the spread of vancomycin-resistant *Staphylococcus aureus* (VRSA) is reported to be progressing overseas [17,18]. Therefore, the investigation of the distribution of VRSA in the water environment as well as MRSA and the assessment of the environmental risk need to be required in the near future. Our results support the need for further, conclusive research performed by taking experimental, technical, regional customs, bias, and unknown factors into consideration.

Mass flux-based analysis of the contributions of *S. aureus* and MRSA from the wastewater to the rivers showed less impact in the targeted area in the present survey. However, there have been many reports pointing out the impact of wastewater discharged into the water environment. In fact, several studies have shown that the contribution was a maximum of several tens of % to 71%, considering all of the medical institutions located within the area and the impact of hospital effluent on the environment [31,66,67,68,69]. Further, it is noteworthy that the flow rate of the STP in this study accounts for approximately 5% to 8% of the total flow rate of all of the STPs in the river basin [44], but in some cases the contribution rate of the total pollution load of all STPs in the river basin was approximately 10% to 30% [70,71,72]. However, these studies reported the values for micropollutants such as pharmaceuticals and personal care products, but data for microorganisms were currently not available.

Considering that these wastewaters are composed of substances such as domestic wastewater and hospital effluent generated from homes and businesses, it is likely that domestic wastewater as well as hospital wastewater is involved as the source of these microbes. This suggests that the current spread of MRSA in Japan is not limited to medical institutions, but may be widespread throughout the city as already mentioned [16,17,18]. Although the degree of pollutant loading from hospital effluent to STP influent varies greatly from region to region and country to country [73,74,75,76,77,78], our results support the need for further conclusive research by taking regional customs, bias, and unknown factors into consideration. In addition, due to the presence of a wide variety of live microorganisms in activated sludge in the biological treatment reactors at STPs [79], there have been concerns that AMRB could result in a pool of AMRB through zygotic transmission or transformation [80,81,82]. One of the measures to overcome these challenges is to introduce advanced water treatment systems such as ozonation [83,84,85], membrane processes [86,87], UV [64,88], electrochemical [89,90], and peracetic acid [91,92] treatments that may effectively decrease the levels of these new environmental pollutants in the wastewater discharged into the river environment.

Finally, the results of the QMRA are considered to provide useful and highly important information in terms of being able to quantitatively visualize the health risks of AMRB to humans, which have been difficult to assess in the past, and the required reduction levels. On the other hand, as noted in the Materials and methods section, the QMRA model used in this study was based on the latest but limited knowledge and data. Therefore, it should be noted that improvements are necessary in the future, as findings accumulate and the model is updated. Furthermore, it is also important to consider the use of QMRA by calculating the inactivation rate of MRSA by the actual water treatment process.

## 5. Conclusions

The present research showed the distribution and potential human health risks of *S. aureus* and MRSA from hospital effluent to the river water in a sub-catchment of the Yodo River Basin, Japan. *S. aureus* and MRSA were detected in both the wastewater and river water, and the abundances of MRSA in the wastewater were more than 90%. The higher profiles were also observed in river water, and these were generally consistent with the current prevalence of MRSA in clinical settings in Japan. QMRA showed that to achieve below health benchmarks, a 1.7 log_10_ inactivation for infection and 2.9 log_10_ inactivation for DALYs were required in the drinking water purification process.

These findings suggest the importance of reducing or inactivating *S. aureus* and MRSA before the effluent is discharged into rivers and during the drinking-water purification process to minimize the environmental pollution posed by AMRB in water environments. The overall results provide new insights into preventing the environmental risks associated with the prevalence of MRSA and infectious diseases originating from aquatic environments, and contribute towards the safety of water environments and human health. To our knowledge, this is the first report of the detailed evaluation of the occurrence and environmental health assessment of *S. aureus* and MRSA from hospital effluent, to STP wastewater, and finally to river water at the basin level.

## Figures and Tables

**Table 1:** Occurrence of *Staphylococcus aureus* (*S. aureus*) and methicillin-resistant *Staphylococcus aureus* (MRSA) in hospital effluent, elderly healthcare facility effluent, sewage treatment plant (STP) influent, STP secondary effluent, STP effluent, and river water (*: The river water as a drinking water source was supplied to the study area and neighboring regions, N.D.: Not detected).

Bacteria	Sample Type	Bacteria Counts (CFU/mL)			
		Mean (SD)	Median	Maximum	Minimum
*Staphylococcus aureus* (*S. aureus*)	River (drinking water source) *	6 (2)	6	8	4
	River	13 (7)	14	19	3
	STP effluent	16 (11)	13	31	6
	STP secondary effluent	9 (9)	7	21	2
	STP influent	124 (83)	115	232	35
	Elderly healthcare facility effluent	39 (43)	33	85	N.D.
	Hospital effluent	31 (18)	33	47	12
Methicillin-resistant *Staphylococcus aureus* (MRSA)	River (drinking water source) *	5 (1)	5	6	4
	River	11 (6)	13	17	3
	STP effluent	13 (9)	10	25	6
	STP secondary effluent	8 (7)	6	17	2
	STP influent	117 (78)	108	219	33
	Elderly healthcare facility effluent	36 (39)	30	77	N.D.
	Hospital effluent	29 (17)	30	46	12

**Table 2:** Composition ratio of *Staphylococcus aureus* (*S. aureus*) and methicillin-resistant *Staphylococcus aureus* (MRSA) in each type of water sample.

Sample	Mean Mass Flux (CFU/day)	
	*Staphylococcus aureus* (*S. aureus*)	Methicillin-Resistant *Staphylococcus aureus* (MRSA)
River (drinking water source)	1.1 × 10^14^	8.9 × 10^13^
River	3.5 × 10^13^	3.2 × 10^13^
STP effluent	3.0 × 10^12^	2.4 × 10^12^
STP secondary effluent	1.7 × 10^12^	1.5 × 10^12^
STP influent	2.4 × 10^13^	2.2 × 10^13^
Hospital effluent	1.4 × 10^10^	1.3 × 10^10^

**Table 3:** Contributions of *Staphylococcus aureus* (*S. aureus*) and methicillin-resistant *Staphylococcus aureus* (MRSA) in each type of water sample.

Bacteria	Contribution of Hospital Effluent (% of Total STP Influent)			Contribution of STP Effluent (% of Total River Water)			Contribution of STP Effluent (% of Total River Water (Drinking Water Source))		
	Mean	Maximum	Minimum	Mean	Maximum	Minimum	Mean	Maximum	Minimum
*Staphylococcus aureus* (*S. aureus*)	0.1	0.2	<0.1	14.8	24.7	4.9	4.0	6.8	2.1
Methicillin-resistant *Staphylococcus aureus* (MRSA)	0.1	0.2	<0.1	14.4	25.4	3.9	4.0	7.4	2.1

**Table 4:** Risks associated with direct use of river water.

Risk Unit	Percentile	Total	Abundance of Methicillin-Resistant *Staphylococcus aureus* (MRSA)
Infection (ppy)	50th	4.6 × 10^−3^	98.9%
	95th	4.8 × 10^−3^	98.9%
DALYs (ppy)	50th	7.5 × 10^−4^	99.5%
	95th	7.8 × 10^−4^	99.5%

## Abbreviations

AMRB	antimicrobial-resistant bacteria
CFU	colony-forming units
DALY	disability-adjusted life year
JANIS	Japan nosocomial infections surveillance
MRSA	methicillin-resistant *Staphylococcus aureus*
MSSA	methicillin-susceptible *Staphylococcus aureus*
N.D.	not detected
QMRA	quantitative microbial risk assessment
*S. aureus*	*Staphylococcus aureus*
SD	standard deviation
STP	sewage treatment plant
WHO	World Health Organization
